# EPIDEMIOLOGY OF POST-TRAUMATIC SPINAL CORD INJURY IN A TERTIARY HOSPITAL

**DOI:** 10.1590/1413-785220233105e264492

**Published:** 2023-10-23

**Authors:** VAGNER CLAYTON DE PAIVA, CAMILO VELLOSO NUNES, CAIO VILLELA ANTONIALLI, PEDRO HENRIQUE CALEGARI MORAES, GUILHERME AUGUSTO FOIZER, IURI TOMAZ DE VASCONCELOS, SERGIO SAN JUAN DERTKIGIL, ALBERTO CLIQUET JUNIOR, JOÃO BATISTA DE MIRANDA

**Affiliations:** 1 Universidade Estadual de Campinas, Faculdade de Ciências Médicas, Departamento de Ortopedia e Traumatologia, Campinas, SP, Brazil.; 2 Instituto de Assistência Médica ao Servidor Público Estadual de São Paulo, Departamento de Ortopedia e Traumatologia, São Paulo, SP, Brazil.; 3 Universidade Estadual de Campinas, Faculdade de Ciências Médicas, Serviço de Radiologia, Campinas, SP, Brazil.

**Keywords:** Epidemiology, Spinal Cord Injuries, Paraplegia, Quadriplegia, Epidemiologia, Traumatismos da Medula Espinal, Paraplegia, Tetraplegia

## Abstract

**Objective:**

to outline the profile of risk groups for spinal cord injury (SCI) at the Hospital de Clinicas de Campinas by an epidemiological survey of 41 patients with SCI.

**Methods:**

Data from patients with SCI were collected and analyzed: demographic data, level of neurological injury, visual analogue scale (VAS), and the current American Spinal Injury Association (ASIA) impairment scale (AIS), using questionnaires, medical records, and imaging tests. Fisher’s exact test was used to assess the relationship between categorical variables, Spearman’s correlation coefficient was used for numerical variables, and the Mann-Whitney and Kruskal-Wallis tests were used to analyze the relationship between categorical and numerical variables, with significance level of 5%.

**Results:**

There was a prevalence of 82.9% of men, a mean age of 26.5 years, and traffic accidents as the cause of SCI in 56.1% of cases.

**Conclusion:**

Results suggest the importance of SCI prevention campaigns directed at this population. ***Level of Evidence II, Retrospective Study.***

## RESUMO

**Objetivo:**

Traçar o perfil dos grupos de risco para trauma raquimedular (TRM) do Hospital das Clínicas de Campinas através de levantamento epidemiológico de 41 pacientes vítimas de TRM.

**Métodos:**

Foram coletados e analisados dados demográficos, nível da lesão neurológica, escala visual analógica (EVA) e American Spinal Injury Association impairment scale (AIS) atuais, através da aplicação de questionários, análise de prontuários e de exames de imagem. Para avaliar a relação entre as variáveis categóricas foi utilizado o teste exato de Fisher; para as variáveis numéricas foi utilizado o coeficiente de correlação de Spearman; e para a análise da relação entre variáveis categóricas e numéricas foram utilizados os testes de Mann-Whitney e Kruskal-Wallis, adotando nível de significância de 5%.

**Resultados:**

Houve prevalência de 82,9% do sexo masculino, média de idade de 26,5 anos e de 56,1% casos de TRM causados por acidente automobilístico.

**Conclusão:**

Os resultados sugerem a importância da realização de campanhas de prevenção ao TRM voltadas para essa população. **Nível de Evidência II, Estudo Retrospectivo.**

## INTRODUCTION

Spinal cord injury (SCI) includes any type of injury to the spine (bone, ligament, spinal cord, disc, vascular, or radicular)^1^ that leads to permanent or temporary dysfunction of the spinal cord and consequent neurological, motor, sensory, and/or autonomic deficits,^2^ affecting not only physical but also mental and social health of individuals, and transforming young productive individuals into people who require specialized, high-cost care.^3^

A recent meta-analysis reviewed the worldwide incidence of SCI, reaching 105 new cases per million inhabitants, with a higher incidence in developing countries.^2^ Traffic accidents and falls from height were the main causes of SCI in the studies.^4^ In Europe, estimates show 16 new cases per million, less than half the incidence in the United States (US).^5^ ^),(^ ^6^

The incidence of SCI in the US is 38 new cases per million inhabitants per year, or 10,000 new cases per year, of which 4,000 die before reaching hospital and 1,000 during hospitalization. The highest incidence is among individuals aged 20 to 24 years, of whom 65% are under 35 and 80% are men. Motor vehicle accidents correspond to 50% of cases, followed by falls, with 15% to 20%.^7^

Knowledge of the epidemiology of SCI is essential for proposing preventive measures and concentrating technical and human resources in reference services for the care and treatment of these patients.^8^

Thus, this study aimed to perform an epidemiological survey of patients with SCI at the Hospital de Clinicas de Campinas (HC/UNICAMP), in order to outline a profile of risk groups for SCI, allowing not only treatment and rehabilitation, but also primary prevention.

## METHODS

### Data collection

From May 21, 2018 to May 15, 2019, data were collected from 41 patients with SCI who underwent different treatments in different Brazilian hospitals and were followed up at the Ambulatorio de Reabilitaçao Raquimedular, Departamento de Ortopedia e Traumatologia do Hospital de Clinicas da Universidade Estadual de Campinas (UNICAMP), Brazil.

Medical questionnaires were applied to all patients and medical records and imaging tests (X-rays, CT scans, and MRI scans) were analyzed. Demographic data and information on the level of SCI and the treatment performed at the time of the trauma were collected. The visual analog scale (VAS) and the current American Spinal Injury Association (ASIA) impairment scale (AIS) were used to evaluate the neurological level.^9^

Patients who did not agree to participate in the study and who did not have adequate imaging tests to define the diagnosis of trauma were excluded.

The procedures of this study are in accordance with the 1995 Declaration of Helsinki. The study was duly approved by the institution’s Research Ethics Committee (CAAE: 84859717.5.0000.5404). All patients voluntarily participated in the study and signed the informed consent form.

### Statistical analysis

To describe the profile of the sample, frequency tables were prepared for the categorical variables, with absolute frequency (n) and percentage (%) values, and descriptive statistics for the numerical variables, with mean, standard deviation, minimum, maximum, and median values.

Fisher’s exact test was used to assess the relationship between categorical variables. Spearman’s correlation coefficient was used to assess the relationship between the numerical variables. The Mann-Whitney and Kruskal-Wallis tests were used to assess the relationship between categorical and numerical variables, with a significance level of 5%.^10^

## RESULTS

This study included 41 patients, 34 men (83%) and seven women (17%). The mean age at the time of the trauma was 26.5 years (11-46 years) and the mean body mass index (BMI) was 23.6 kg/m^2^ (15.8-32.3 kg/m^2^). Table 1 presents the distribution of the study population by sex.

**Table 1:** Population distribution by sex.

Sex	Frequency	%
Women	7	17.1
Men	34	82.9

The population included 23 (56.1%) victims of traffic accidents, four (9.8%) victims of falls from height, seven (17.1%) victims of shallow water diving, and seven (17.1%) victims of stab wounds (SW) or gunshot wounds (GSW). Table 2 presents the distribution of SCI by causes.

**Table 2:** Distribution of spinal cord trauma by causes.

Category	Frequency	%
Traffic accident	23	56.1
SW/GSW	7	17.1
Shallow water diving	7	17.1
Fall from height	4	9.7

SW/GSW: stab wound/gunshot wound

The mean age at the time of the assessment was 39.4 ± 11.2 (17-65) years. The mean time of assessment after initial trauma was 151.3 ± 83.1 (13-276) months. The mean age of patients at SCI was 26.5 ± 8.3 (11-46) years. Their mean weight was 72.2 ± 13.7 (50-113) kg. The mean height was 1.75 ± 0.1 (1.6-1.9) m. The mean BMI was 23.6 ± 4 (15.9-32.3) kg/m^2^. The mean VAS score was 2.4 ± 2 (0-7). Table 3 shows the demographic data of the study population.

**Table 3:** Demographic data of the study population.

Variable	Mean ± SD	Minimum-Maximum
Current age (years old)	39.4 ± 11.2	17-65
Time of SCI (months)	151.3 ± 83.1	13-276
Age at SCI (years old)	26.5 ± 8.3	11-46
Weight (kg)	72.2 ± 13.7	50-113
Height (m)	1.75 ± 0.1	1.6-1.9
BMI (kg/m^2^)	23.6 ± 4	15.9-32.3
VAS	2.4 ± 2	0-7

SCI: spinal cord trauma; BMI: body mass index; VAS: visual analog scale.

In total, 18 (43.9%) victims had cervical trauma and 23 (56.1%) had thoracic trauma.

Moreover, 21 patients had paraplegia (51.2%) while 20 had quadriplegia (48.8%). Table 4 presents the frequency of paraplegia and quadriplegia in the study population.

**Table 4:** Frequency of paraplegia and quadriplegia in the study population.

Category	Frequency	%
Paraplegia	21	51.2
Quadriplegia	20	48.8

Our population included three AIS 4 patients (7.3%), 37 AIS 5 patients (90.2%) and one AIS 6 patient (2.4%). Table 5 shows details of the neurological assessment of the study population.

**Table 5:** American Spinal Injury Association impairment scale classification of the study population.

AIS	Frequency	%
4	3	7.3
5	37	90.2

AIS: ASIA impairment scale.

In total, 10 patients (24.4%) underwent conservative treatment and 31 (75.6%) underwent surgical treatment. Table 6 shows that of the patients treated surgically, 10 were operated by anterior approach (24.4%), 19 by posterior approach (46.3%), and two by double approach (4.9%).

**Table 6:** Surgical treatments performed.

Category	Frequency	%
Anterior approach	10	32.3
Posterior approach	19	61.3
Double approach	2	6.4

## DISCUSSION

In Brazil, estimates show the occurrence of 40 new cases of SCI per million inhabitants, representing about 8,000 new cases a year, with a high cost to the health system. The most frequent causes are SW/GSW, traffic accidents, shallow water diving, and falls from height. It occurs predominantly in young adults, and 60% of victims are aged 10 to 30 years, in a ratio of four men to one woman.^3^ ^),(^ ^11^

A recent systematic review analyzed 10 studies on the epidemiology of SCI in Brazil, published from 2010 to 2016. The highest incidence of SCI was among men, young adults, and individuals with low schooling levels, and the most common causes were traffic accidents, falls from height, and SW/GSW.^12^

A recent study conducted in the US used the National Electronic Injury Surveillance System to assess cervical and thoracic spine fractures from 2007 to 2016, stratified by demographic data such as sex, age, and ethnicity. Of the 131,176 fractures identified, 95.4% were in the thoracic spine, 4.7% in the cervical spine, and 1.2% involved both. Moreover, 91.1% were single-level lesions and 8.9% affected multiple levels. The most common age was 20 to 29 years.^13^

A population-based prospective cohort study showed that the cost per patient was almost U$ 200,000.00 during the first two years after the injury, including home care, medical services, and secondary complications. In the US, about 15% of patients with spinal trauma are likely to have neurological impairment.^14^

A meta-analysis of 64 studies on the epidemiology of SCI in 28 developing countries showed an incidence of 25.5 cases per million inhabitants per year, ranging from 2.1 to 130.7/million/year. Men account for 82.8% of cases, with a mean age of 32.4 years. The main causes of SCI were traffic accidents (41.4%) and falls (34.9%). Complete SCI occurred in 56.5% of cases, and paraplegia (58.7%) was more common than quadriplegia (40.6%).^15^

A Brazilian study showed that the main causes of SCI were traffic accidents, falls from height, SW/GSW, and diving, and the most affected region was the dorsal spine. Most patients progressed to Frankel A.^16^ These results are similar to the findings of our study.

In a 2018 study with 2,076 patients from a network of rehabilitation hospitals in Brazil, 83% were men, the mean age was 31 years, 67.7% had paraplegia, and the main cause was traffic accident (43.7%), followed by GSW (28.4%).^17^

A study conducted in a tertiary hospital in Sao Paulo evaluated 515 patients with SCI, of whom 85.6% were men, the mean age was 39.4 years, the main cause was fall from height (47%), and 52.9% of patients were classified as Frankel A.^3^

A retrospective study analyzed the epidemiology of SCI in a public hospital in Joinville, Santa Catarina, and showed a prevalence of 87% of men. A total of 47.8% of the injuries were caused by motor vehicle accidents and 26.1% by GSW.^18^

The data from the aforementioned study were similar to the findings of our study, which showed a prevalence of 82.9% of men, a mean age at SCI of 26.5 years, and traffic accidents as the cause of SCI in 56.1% of cases.

Clinical trials and case reports suggest that early treatment can improve the neurological recovery of these patients and prevent further spinal cord damage.^19^

SCI is an important cause of morbidity, affecting young adults and producing extremely severe socioeconomic consequences, with life-long costs for rehabilitation and loss of productivity for the patient. Mortality is higher the older and the worse the neurological status of the patient.^20^

## CONCLUSION

Due to the high morbidity of SCI and its prevalence mainly in young adult men, SCI prevention campaigns directed at this population are essential.
